# Editorial: Dynamic contrast-enhanced imaging: technology progress and clinical application in oncology

**DOI:** 10.3389/fonc.2025.1681143

**Published:** 2025-09-10

**Authors:** Zujun Hou, Baowei Fei, Jingliang Cheng

**Affiliations:** ^1^ College of Chemistry and Chemical Engineering, Nanjing University, Nanjing, China; ^2^ Department of Bioengineering, University of Texas at Dallas, Dallas, TX, United States; ^3^ Department of Radiology, University of Texas (UT) Southwestern Medical Center, Dallas, TX, United States; ^4^ Department of Magnetic Resonance Imaging (MRI), First Affiliated Hospital of Zhengzhou University, Zhengzhou, China

**Keywords:** Advanced DCE, tracer kinetic modelling, tissue microenvironment, quantitative imaging biomarker, oncological imaging

In the past a few decades, tremendous evidences have been emerged to drive the evolution of radiology from a qualitative discipline towards a quantitative modern science (1–4), of which dynamic contrast-enhanced (DCE) imaging has been of extensive research interest in oncological imaging for its ability in the non-invasive characterization of microvascular information of tumour from imaging signal (5, 6). The derived quantitative imaging features can be an indicator of normal biological or pathogenic process, surrogated to a clinically significant endpoint, and of great value in a wide variety of clinical problems, such as tumour grade correlation, prognosis prediction, therapeutic effect assessment, treatment response diagnosis, etc (7–9). The results have been reviewed by the working group of RECIST (10, 11), though the technology was not recommended to be included in the revised guideline, namely RECIST 1.1, due to insufficient evidence at that time. Nevertheless, remarkable progress has been made on the modelling of tracer kinetics, as is essential in DCE theory, from one compartment to two compartments, from homogeneous compartment to distributed compartment, from mixed transportation to separate account of blood flow and vessel wall permeability, and such representative models include Brix’s two-compartment model (Brix), tissue homogeneity model (TH) and distributed parameter model (DP) (12, 13). Technique advancement has spurred renewed interest of DCE in clinical investigation, paving the way towards a more comprehensive assessment of tissue microcirculation.

This Research Topic highlights multiple technology advancements and their clinical applications. One important application of DCE lies in distinguishing tumour recurrence from treatment-induced changes in brain tumour patients receiving radiation therapy or concurrent temozolomide chemotherapy after surgery. An investigation was presented recently by applying advanced DCE models to the differential diagnosis of glioma recurrence and treatment response (14), where mean transit time (MTT) by DP attained the best performance with area under the receiver operating characteristic (ROC) curve (AUC) 0.88 when compared with Brix, TH and extended Tofts model (ETM). In this Research Topic, Zhou et al. appraised issues on glioma studies using conventional tracer kinetic models (TKMs), such as Tofts or ETM model, highlighted advancement of DCE imaging techniques and provided insights on the clinical value of glioma management using more advanced DCE models with standardization of protocol design and data post-processing. The ability of accurate differentiation between tumour recurrence and treatment-induced changes is critical in the subsequent treatment planning. In particular, when steep dose gradients in radiation therapy are commonplace, it is imperative for the radiation to be delivered as precisely as possible to reduce organ-at-risk (OAR) constraint violations and mean OAR doses (15).

In PI-RADS v2.1 (16), DCE-MRI is mandated to be interpreted in conjunction with T2-weighted imaging and diffusion weighted imaging, though the major role of DCE-MRI in PI-RADS v2.1 has been downgraded to a qualitative binary classifier in the lesion within the peripheral zone of the prostate only when differentiating between a PI-RADS score of 3 and 4. In this Research Topic, Zhang et al. pointed out that only qualitative (uptake and washout curve pattern with limited imaging time points) or semi-quantitative methodology was reviewed in PI-RADS v2.1, and the downgrading of DCE-MRI in PI-RADS could possibly be related to variation in DCE data acquisition and analysis, where visual examination by radiologists was the dominant method for DCE image analysis. Moreover, Zhang et al. investigated the quantitative DCE parameters from different DCE models to discriminate prostate cancer (PCa) and normal tissue and demonstrated that most parameters showed significant differences, and all models presented good performance, with one or more parameters attaining AUC>0.80. In the recently published Prostate Imaging for Recurrence Reporting (PI-RR) system (17), the value of DCE was recognized in detecting local PCa recurrence with biochemical relapse after local treatment with curative intent, where the PI-RR assessment after radiation therapy is mainly derived from the DWI and DCE sequences (of which DCE would be of particular importance when DWI could be subject to susceptibility artefacts after low-dose-rate brachytherapy), and the final PI-RR assessment score after radical prostatectomy is generated using the individual DWI and DCE sequences, with DCE being the dominant sequence. Nevertheless, the value of quantitative parameters using advanced DCE models in PI-RR remains to be elucidated.

In BI-RADS, DCE-MRI is specifically utilized for kinetic assessment of changes in signal intensity over time with unique descriptors for the initial and delayed phases of contrast kinetics (18), where abnormal enhancement (unique and separate from the background parenchymal enhancement) is described based on morphology, distribution, and kinetics, and it is mandated that masses that enhance and are identified or non-mass enhancement on an initial MRI examination should undergo assessment based on morphology and kinetics in the follow-up MRI examination. In this Research Topic, Jiang et al. analysed the semi-quantitative parameters derived from DCE-MRI in 21 patients with type II time intensity curve (TIC) tumours, and demonstrated that time to peak showed significant difference between benign and malignant classification of masses with type II TIC curves. Mou et al. presented the imaging characteristics of malignant glomus tumour in breast, which is very rare in breast cancer and has never been reported before. Recently, Sallauka et al. (19) reviewed the latest advancements in breast cancer recurrence markers, and identified nuclear grade, microenvironment heterogeneity, estrogen receptor, androgen receptor, human epidermal growth factor receptor 2, Ki-67 antigen, as the most significant histopathological markers of breast cancer recurrence. Quantitative parameters derived from ETM in breast cancer has been shown that mean Ktrans or Kep was associated with high histologic and high nuclear grade or hormone receptor negativity (20). No report has been presented thus far on the investigation of the diagnostic or prognostic value of advanced DCE techniques as potentially minimally invasive or minimally intrusive markers for breast cancer recurrence detection.

In this Research Topic, Xu et al. evaluated clinical presentation and imaging characteristics of

leiomyosarcomas of the inferior vena cava using contrast-enhanced CT, ultrasonography, MRI, and identified that the detection of a heterogeneous mass with progressive enhancement along the inferior vena cava which might facilitate early and accurate pre-operative diagnosis. Recently, Gao et al. assessed the efficacy of various DCE models for categorizing benign and malignant soft tissue tumours and demonstrated that all DCE models accurately distinguished between such lesions, and DP attained the highest AUC (21).

With the progress in DCE theory and imaging facility, as well as more readily available advanced DCE imaging software, increasing evidence demonstrates the advantage of modern DCE technology in characterization of tumour microenvironment. Nevertheless, sustained clinical trials in large scale and multiple centers are necessary to establish the reproducibility of technique and enable the full potential of DCE as quantitative imaging biomarkers being realized in routine clinical practice. It is anticipated that related guidelines will continue to evolve with the persistent development of DCE technology.
